# Neuroprotective Effects of Polyphenol-Rich Corinthian Currant Against a Rotenone Parkinson’s Disease Model: Mitigation of MAO-B and Pro-Inflammatory Cytokines Upregulation in Motor and Limbic Brain Regions

**DOI:** 10.3390/antiox15070906

**Published:** 2026-07-22

**Authors:** Eleni Fanarioti, Martha Tsarouchi, Christina Mountaki, Vaios Karathanos, Angeliki Chroni, Catherine R. Dermon

**Affiliations:** 1 Human and Animal Physiology Laboratory, Department of Biology, University of Patras, 26504 Patras, Greece; efanariot@upatras.gr (E.F.); martha.tsarouchi@gmail.com (M.T.); 2 Institute of Biosciences and Applications, National Center for Scientific Research “Demokritos”, Agia Paraskevi, 15341 Athens, Greece; xmountaki@outlook.com (C.M.); achroni@bio.demokritos.gr (A.C.); 3 Laboratory of Chemistry-Biochemistry-Physical Chemistry of Foods, Department of Dietetics and Nutrition, Harokopio University, 17676 Kallithea, Greece; vkarath@hua.gr; 4 Agricultural Cooperatives’ Union of Aeghion, Corinthou 201, 25100 Aeghion, Greece

**Keywords:** Parkinsonian syndrome, neuroinflammation, IL-1β, TNF-α, MAO-B, NF-κB, AChE, substantia nigra pars compacta, hippocampus, prefrontal cortex, microglia

## Abstract

Parkinson’s disease (PD) is a progressive neurodegenerative disorder of motor function, while at advanced stages it includes memory deficits and neuropsychiatric conditions. It is characterized primarily by the loss of dopaminergic neurons in the substantia nigra pars compacta (SNpc) and reduced dopamine levels in the striatum. Accumulating evidence highlights the role of polyphenols against oxidative stress and neuroinflammatory processes, underlying key factors of PD pathogenesis. The present study used a rotenone rat model to determine the involvement of inflammatory cytokines in the basal ganglia, hippocampus, basolateral amygdala, and prefrontal cortex and to evaluate the potential of black Corinthian currant, a fruit abundant in antioxidant polyphenols, to attenuate brain inflammatory responses. Rotenone is known to trigger oxidative stress by inhibiting mitochondrial complex I and thus induce dopaminergic neurodegeneration, further amplified by inflammation. Our findings clearly demonstrated that rotenone treatment resulted in significant increases in pro-inflammatory cytokines as well as MAO-B expression within the rat brain motor and limbic regions. Importantly, dietary supplementation with black Corinthian currant mitigated rotenone-induced overexpression of IL-1β, TNFα, and MAO-B. Moreover, double immunofluorescence suggested the microglial localization of these inflammatory markers in SNpc. Overall, our data highlight the neuroprotective potential of dietary Corinthian currant polyphenols, modulating glial-mediated neuroinflammation in a rotenone PD model.

## 1. Introduction

Parkinson’s disease (PD) is characterized by the loss of dopaminergic neurons in the substantia nigra pars compacta (SNpc), which leads to the dysfunction of dopaminergic transmission in the nigrostriatal pathway [1]. However, PD etiology is multifactorial, involving mitochondrial dysfunction [2] and neuroinflammation, including microglial activation [3]. Microglia, the resident macrophages of the brain, under physiological conditions, provide neuronal protection through anti-inflammatory processes and neurotrophic factor release [4]. In contrast, pathological microglial activation drives chronic neuroinflammation, which accelerates the degeneration of dopaminergic neurons in the SNpc, thereby promoting the progression of PD [5]. Activated microglia secrete large amounts of pro-inflammatory mediators [6], resulting in neuronal damage, which further activates microglia and leads to perpetual neurodegeneration [7]. Although the role of microglia has been extensively studied in various neurodegenerative disorders [8], the underlying mechanism targeting specifically the loss of dopaminergic neurons in PD is still unclear.

Two main pro-inflammatory mediators, interleukin (IL)-1β and tumor necrosis factor (TNF)-α, are suggested to be involved in regulating neuroinflammation and synaptic transmission [9]. IL-1β, a pro-inflammatory cytokine which exerts several biological effects in both the periphery and the central nervous system, is significantly increased in the striatum of postmortem brains of PD patients [10] and animal models [11,12]. Furthermore, IL-1β expression in the SNpc has been shown to accelerate the neuronal loss of dopaminergic cell bodies, depending on the duration and levels of the cytokine expression [11,12]. In addition, the upregulation of IL-1β expression has been shown in postmortem non-motor limbic and cortical brain areas of PD patients with dementia [13]. Likewise, TNF-α levels are elevated in PD patients and animal models [14,15] and were shown to induce dopaminergic cell loss in SNpc [16]. Activated astrocytes and microglia release TNF-α, which binds to the tumor necrosis factor receptor-1 (TNFR1) on dopaminergic neurons to trigger apoptosis, promoting neuroinflammation and neurodegeneration [17]. Importantly, due to the persistent microglial activation, the NF-κB pathway is constantly stimulated [18]. NF-κB is a key transcription factor involved in microglia activation, driving the neuroinflammatory processes related to the pathology of neurodegenerative diseases like PD [19,20].

Moreover, dopamine levels are highly regulated for optimal neurotransmission and to prevent cellular toxicity. Specifically, cytosolic dopamine is degraded, via mitochondrial monoamine oxidase (MAO), into the toxic intermediate DOPAL, which is quickly converted into the non-toxic metabolite DOPAC, which can rapidly exit the cell. However, in PD, monoamine oxidase-B (MAO-B) is significantly upregulated within reactive astrocytes in the substantia nigra [21], rapidly breaking down dopamine, accelerating the formation of DOPAL, which in turn amplifies the neuroinflammatory cascade and ultimately promotes disease progression. It should be noted that inhibiting neuroinflammation or MAO-B activity is a promising therapeutic strategy to increase dopamine levels and potentially offer neuroprotection.

Accumulating evidence indicates that natural plant-derived polyphenols have a beneficial action in neurodegenerative diseases by targeting inflammatory processes (e.g., genes, signal transduction pathways), thus mitigating neuroinflammation and enhancing neuroprotection [22,23]. Corinthian currant (*Vitis vinifera*) is a traditional Greek vine product that naturally dries in the sun and has a high content of polar phenol classes, such as flavones, flavonols, flavanols, flavanons, benzoic acids, phenylacetic acids, cinnamic acids, and anthocyanins [24]. We have previously shown that the polar phenols of Corinthian currant, such as quercetin, isoquercetin, rutin and isorhamnetin, can cross the blood–brain barrier (BBB), accumulate in different brain regions, attenuating motor deficits, offering protection to the nigrostriatal pathway by reducing the loss of dopaminergic cells in SNpc and increasing BDNF expression in the rotenone PD model [25,26]. In addition, several phenolic compounds have been suggested to reduce the levels of pro-inflammatory mediators such as TNF-α, IL-1β, and NF-κB [27,28], as well as astrocyte and microglia activation [29,30], in neurological disorders and experimentally induced cerebral ischemia.

Considering all the above, the aim of the present study was to question the role of dietary polyphenolic compounds in neuroinflammatory processes in a rotenone rat PD model. Rotenone induces degeneration of the dopaminergic nigrostriatal pathway by the inhibition of the mitochondrial complex I, which generates superoxide anions, through electron leakage [31]. This neurodegenerative cascade is suggested to be further amplified by inflammation targeting primarily the dopaminergic neurons, that are characterized by high baseline metabolic demands, high endogenous iron content, and low glutathione levels. For this, we examined the rotenone-induced neuroinflammatory processes in the nigrostriatal pathway and in the corticolimbic system that has been shown to be also significantly affected by rotenone toxicity [32]. In addition, we determined the possible mitigating effects of Corinthian currant consumption, focusing on the key pro-inflammatory cytokines (IL-1β and TNF-α), as well as MAO-B and AChE, by quantitative immunohistochemistry, double immunofluorescent labeling, and the ELISA method.

## 2. Materials and Methods

### 2.1. Animals

Male Wistar rats (*n* = 48) weighing 200 to 250 g at the beginning of the experimental protocol (postnatal days 60 to 70) were housed individually at the Laboratory of Experimental Animals, University of Patras, under a 12-h-light/dark cycle with free access to food and water. Animal experiments were conducted and reported in accordance with ARRIVE guidelines, EU Directive 2010/63/EU of the European Parliament and of the Council of 22 September 2010 on the protection of animals used for scientific purposes. *Official Journal of the European Union*, L 276, 20.10.2010, pp. 33–79. Publisher: Publications Office of the European Union, Luxembourg, Luxembourg 2010, for laboratory animal care and use, and were approved by the University of Patras ethics committee and by the Veterinary Administration of the Prefecture of Achaia, Greece (Protocol number: 187526/625/26-06-2018). Efforts were made to minimize animal suffering and to reduce the number of animals used.

### 2.2. Experimental Design

Rotenone treatment protocol and Corinthian currant supplementary diet are precisely described in our previously published studies [25,32]. At the beginning of the experimental procedures, all rats were handled for a 10-day period in order to get familiarized with the researchers, and were randomly assigned to 4 experimental groups (Rotenone, Rotenone_currant, Control, Control_currant). Rats were injected subcutaneously (s.c) 1 mL/kg weight, daily for 28 days, either with Rotenone (Cat #R8875, Sigma, St. Louis, MO, USA), 2.5 mg/kg of body weight [33] suspended in vehicle solution containing 1% dimethylsulfoxide (Sigma, St. Louis, MO, USA) in sunflower oil (Rotenone group), or with vehicle (Control group). In order to ensure a uniform suspension, rotenone was vortexed thoroughly just before injection. Corinthian currant diet supplementation was applied in the morning (entire fruit, 3% of the daily food intake) starting 10 days before rotenone/vehicle s.c. treatment and was consumed per se in addition to conventional food for a total of a 38-day period (10 days before and throughout the s.c. treatment) (Rotenone_currant and Control_currant groups) [25]. At the end of the experimental protocol, rats were tested for motor, exploratory, and anxiety-like behaviors as previously described [25,32].

High-quality sun-dried Corinthian currants (*Vitis vinifera* L. var *Apyrena*), namely Vostizza currants [24], that hold a protected designation of origin (PDO) name, were provided by the Agricultural Cooperatives’ Union of Aeghion, Greece. The currants were processed and stored according to the principles of Hazard Analysis Critical Control Point (HACCP) and routinely underwent Quality Control, including physical, chemical, and microbiological parameters. It should be noted that in our previous studies, we identified and quantified the polar phenolic compounds in Corinthian currants and in commercial standard pellets used in the animals’ diet in the present study. Moreover, we have determined their brain regional accumulation by chromatographic analysis and Mass Spectrometry (UHPLC-MS) [25,26]. Briefly, among the polar phenol compounds identified, Quercetin, Isoquercetin, and Rutin showed high concentrations in Corinthian currants and accumulated in the mesencephalon, striatum, cortex, and hippocampus following the currant-supplemented diet.

The sample size was determined via an a priori power analysis using G*Power software (version 3.0.10; Heinrich-Heine-Universität Düsseldorf, Düsseldorf, Germany). The calculation was based on a medium-to-large effect size (Cohen’s f = 0.71) derived from data on TH+ cell densities comparing the experimental groups (Control vs. Rotenone and Rotenone vs. Rotenone_currant). Setting the statistical significance level at α = 0.05 and the desired statistical power at 0.80 (1-β), this cohort size was verified as sufficient to detect biologically meaningful differences while ensuring group homogeneity and minimizing inter-animal variation.

Rotenone-induced dopaminergic cell loss was verified via TH+ cell density quantification in SNpc. In accordance with our previous findings [25], rotenone treatment significantly reduced TH+ cell densities (the Rotenone group showed a 45.43% decrease in cell density compared to the Control group). Moreover, it was validated that currant consumption effectively mitigated this loss, as evidenced by a significantly lesser reduction of TH cell density in the Rotenone_currant group (by 19.96%). Indeed, currant supplementation resulted in 43.9% higher TH+ cell density in comparison to the rotenone group.

### 2.3. Tissue Preparation for Immunohistochemistry

For immunohistochemistry experiments, rats (*n* = 24) were anesthetized with a combination of ketamine (100 mg/kg) and xylazine (10 mg/kg), transcardially perfused with 0.9% NaCl, followed by ice-cold 4% paraformaldehyde (PFA) in 100 mM phosphate-buffered saline (PBS), pH 7.4. Brains were carefully isolated, cryopreserved, embedded in tissue freezing medium (Jung, Leica Instruments, Instr., Wetzlar Germany) and frozen in 2-methyl-butane. Coronal sections (20 μm thick) were cut at specific coordinates of the rat brain atlas [34], using a cryostat (Leica CM1500 (Wetzlar, Germany)), and immediately stored at −75 °C. Prior to immunostaining, antigen retrieval was performed in citrate buffer at 85 °C for 20 min. Endogenous peroxidase activity was blocked with 0.03% H_2_O_2_ followed by nonspecific serum blocking (1% NHS with 5% BSA, Sigma–Aldrich, Deisenhofen, Germany, and 0.5% Triton X-100 in PBS) for 60 min at RT. Then, sections were incubated overnight at 4 °C with the respective primary antibodies diluted in PBS-T containing 1% BSA and 0.15% NHS; rabbit anti-IL-1β (1:100, ThermoFisher Scientific, Waltham, MA, USA, Cat# P420B), rabbit anti-TNF-α (1:250, Novus Biologicals, Centennial, CO, USA, Cat# NBP1-19532), mouse anti-AChE (1:500, Santa-Cruz Biotechnology, Dallas, TX, USA, Cat# sc-373901) or mouse anti-MAO-B (1:500, Santa-Cruz Biotechnology, Dallas, TX, USA, Cat# sc-515354) in PBS-T with 1% BSA and 0.15% NHS. Sections were then incubated at RT with the appropriate secondary antibodies: either the ready-to-use biotinylated anti-rabbit (R.T.U., Vector, Burlingame, CA, USA, Cat# BP-1100) for 50 min, for IL-1β and TNF-α or the biotinylated horse anti-mouse IgG, component sourced directly from the commercial ABC Vectastain kit (1:200 for 2 h, Vector, Burlingame, CA, USA, Cat# PK6102), for the other primary antibodies) at RT. Subsequently, slides were incubated with the Avidin-Biotin Complex (ABC Vectastain kit (Vector, Burlingame, CA, USA) for 1 h at RT in the dark. Diaminobenzidine (DAB, Vector, Burlingame, CA, USA, Cat# SK4100) was utilized as a chromogen. After rinsing with cold PBS buffer, dehydrating with alcohols, and clearing with xylenes, slides were coverslipped with Entellan (Merck, Darmstadt, Germany).

### 2.4. Double Immunofluorescence Labeling

In order to evaluate the potential co-localization of IL-1β+ with AChE+, MAO-B+, or OX-42+ cells, as well as TNF-α+ with OX-42+, NF-κB+ cells within the SNpc, double-immunolabeling experiments were performed in adjacent sections. Following antigen retrieval, sections were incubated for 18 h at 4 °C with appropriate combinations of the following primary antibodies: rabbit polyclonal anti-IL-1β (1:100, ThermoFisher Scientific, Waltham, MA, USA), mouse monoclonal anti-AChE (1:500, Santa-Cruz Biotechnology, Dallas, TX, USA), mouse monoclonal anti-MAO-B (1:500, Santa-Cruz Biotechnology, Dallas, TX, USA), mouse monoclonal anti-OX-42 (1:200, Invitrogen, Carlsbad, CA, USA, Cat# MA1-81606), rabbit polyclonal anti-TNF-α (1:250, Novus Biologicals, Centennial, CO, USA), or mouse monoclonal anti-NF-κB (1:200, Santa-Cruz Biotechnology, Dallas, TX, USA, Cat# sc-8008). Antibodies were diluted in PBS-T buffer with 5% BSA and 1% NHS. Subsequently, sections were incubated with the secondary antibodies—donkey anti-mouse Alexa Fluor 555 (Invitrogen, Carlsbad, CA, USA, Cat# A-31570) and donkey anti-rabbit Alexa Fluor 488 (Invitrogen, Carlsbad, CA, USA, Cat# A-21206), both diluted 1:400 in PBS-T with 1% BSA and 0.15% NHS)—for 2.5 h at RT in absolute darkness. Finally, the sections were extensively rinsed with PBS and distilled water and coverslipped with the Vectashield hard medium (Vector, USA, Cat# H-1400).

### 2.5. Microscopic Analysis and Quantification

To determine the density of immunoreactive cells, 3 coronal sections per brain region of interest, per animal, for each specific marker studied were analyzed. Quantification was performed within 3 randomly selected square frames, 100 μm^2^ per section, per brain hemisphere (18 frames/brain area/animal), at ×400 magnification with the aid of a camera lucida attached to a light microscope (Optiphot-2 microscope Nikon, Nikon Corporation, Tokyo, Japan). Images were acquired with a Nikon Optiphot 2 microscope, connected to a PC, via a color CCD SONY camera (DXC-950P) (Tokyo, Japan) at ×400 and ×200 (for PFC images) magnification using Scion Image (version 4.0, Scion Corporation, Frederick, MD, USA). To eliminate observational bias, the investigator performing the quantification was blinded to the experimental groups. The specific brain areas of interest were determined using the rat brain atlas [34], at the following coordinates relative to Bregma: striatum (+2.04 to +0.24 mm); SNpc (−4.56 to − 6.24 mm); mPFC and vOFC (+4.68 to +3.50 mm); BLA and hippocampal CA1, CA2, and CA3 (−2.28 to −2.76 mm). Identical anatomical levels were strictly matched across all experimental cohorts to minimize potential sampling bias.

### 2.6. Confocal Microscopy

Double immunofluorescence staining was visualized by a Leica TCS SP8 (Leica Microsystems CMS GmbH) confocal microscope, using the X63 objective. Images were acquired via LASX software (version 3.5.7; Leica Microsystems CMS GmbH, Wetzlar, Germany), maintaining strictly fixed laser intensities and detector settings across all samples to ensure consistency. For the quantification of double-labeled cells, high-resolution images were captured using a X 40 objective lens, defining standardized optical fields of equal area restricted strictly within the anatomical boundaries of the SNpc. Within these predefined fields, the number of IL-1β+ cells co-expressing the microglial CD11b+ (clone OX-42) was determined via manual cell counting on the software- generated multi-channel overlays. To ensure objectivity, the investigator performing the quantification was blinded to the experimental groups.

### 2.7. ELISA

Rats (*n* = 20) were anesthetized with a combination of ketamine (100 mg/kg) and xylazine (10 mg/kg) and subsequently euthanized by rapid decapitation. The striata, mesencephalon, frontal cortex, and hippocampus were promptly dissected according to the rat brain atlas [34], frozen in 2-methyl-butane and stored at −75 °C until the ELISA experiments were performed. The isolated brain tissues were homogenized in ice-cold RIPA lysis buffer at a 1:10 (*w*/*v*) ratio. The RIPA buffer (pH 8.0) contained 50 mM Tris-HCl, 150 mM NaCl, 1% NP-40, 0.5% sodium deoxycholate, 0.1% SDS, and was supplemented with a protease and phosphate inhibitor cocktail (Roche Life Science, Roche Diagnostics GmbH, Mannheim, Germany, Cat# 04693124001). The resulting homogenates were sonicated, incubated on ice for 1 h with vortexing every 10 min, and then centrifuged at 3300 rpm for 5 min at 4 °C to precipitate insoluble material. The supernatants were harvested, and total protein concentrations were quantified using the BCA protein assay (Thermo Fisher Scientific, Waltham, MA, USA, Cat# 23227). Finally, TNF-α levels were determined in homogenate fractions (100 μL per sample) using the rat TNFalpha DuoSet^®^ ELISA kit (Cat#DY510, R&D Systems, Minneapolis, MN, USA), according to the manufacturer’s protocol. All cytokine levels were normalized to the total protein concentration of each respective fraction.

### 2.8. Statistical Analysis

Quantitative data are presented as mean ± SEM. Statistical analyses were conducted using the IBM SPSS Statistics software for Windows, version 29.0 (IBM Corp., Armonk, NY, USA). Statistical comparisons were performed using a two-way analysis of variance (ANOVA) followed by the Bonferroni post hoc test to identify differences in the expression of the inflammatory markers studied (cell immunodensity) and the protein levels between the experimental groups. The factors of variation were rotenone treatment and currant administration as independent variables. Differences were considered significant at *p* < 0.05. *p* values for statistically significant differences are indicated in each figure legend. Graphs were constructed in GraphPad Prism 9.0 (GraphPad Software Inc., San Diego, CA, USA).

## 3. Results

### 3.1. IL-1β and AchE Immunoreactivity Following Rotenone Treatment and Supplementary Diet with Corinthian Currants in Motor Brain Regions

**Figure 1:**
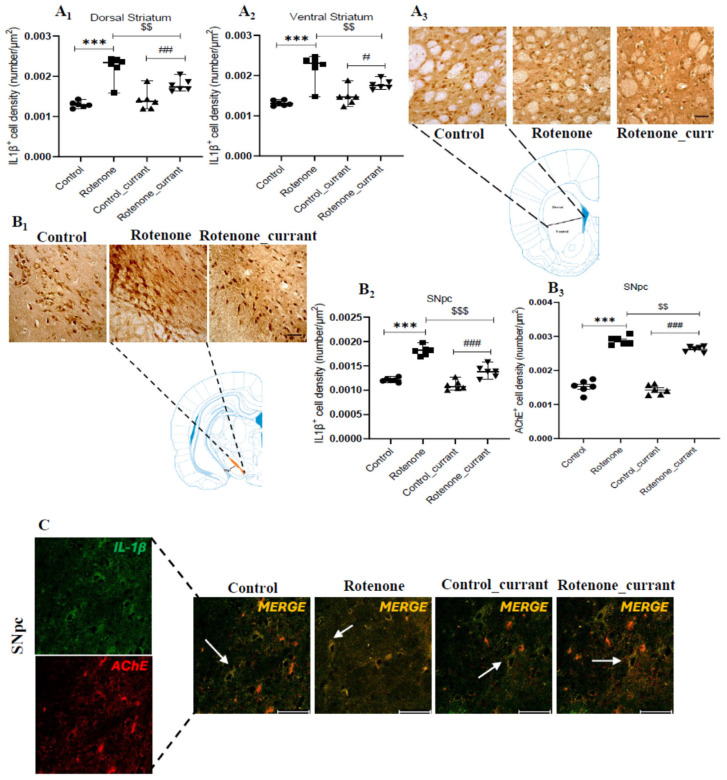
Scatter-dot plots showing the effects of rotenone treatment and Corinthian currant consumption on IL-1β immunodensity in rat (**A_1_**) dorsal and (**A_2_**) ventral striatum. (**A_3_**) Representative photomicrographs of control and rotenone-treated groups in the dorsal striatum. (**B_1_**) IL-1β+ representative photomicrographs of experimental groups in SNpc. Scale bar = 0.05 mm. Schematic diagrams show the indicative coronal levels of striatum subdivisions and SNpc shown in red (modified from [34]). Scatter-dot plots of (**B_2_**) IL-1β and (**B_3_**) AChE immunodensity in SNpc. Data are expressed as mean ± SEM, *n* = 6 per group. *** *p* ≤ 0.001 (Control-Rotenone), ^#^ *p* ≤ 0.05, ^##^ *p* ≤ 0.01, ^###^ *p* ≤ 0.001 (Control_currant-Rotenone_currant), ^$$^ *p* ≤ 0.01, ^$$$^ *p* ≤ 0.001 (Rotenone- Rotenone_currant). (**C**) Indicative confocal immunofluorescent microphotographs showing the colocalization of IL1β+ (green) with AChE+ (red) cells in SNpc of experimental groups. White arrows show indicative double-labeled cells of IL-1β+ and AChE+. Scale bar = 0.05 mm.

***Striatum*:** The rat striatum was divided into dorsal and ventral subdivisions, as shown in Figure 1(A_3_), in order to analyze the immunoreactivity of IL-1β+ cells following the experimental treatment. Two-way ANOVA in the dorsal striatum indicated a significant simple treatment effect (*F*(1, 23) = 51.518, *p* < 0.001) and interaction between the two factors studied (*F*(1, 23) = 9.476, *p* = 0.006), while no significant food main effect was observed (*p* = 0.088). Following Bonferroni post hoc comparisons, statistically significant increases in IL-1β+ cell densities were observed in both rotenone-treated groups compared to their respective controls (C-R: *p* < 0.001; CC-RC: *p* = 0.009). Furthermore, a slight but significant rescue of IL-1β+ cell density in the rotenone group with supplementary Corinthian currant diet was observed compared with the rotenone group that consumed only conventional food (*p* = 0.003). No differences were observed between the two control groups (C-CC: *p* = 0.374). Similar results have also been observed in the ventral striatal subdivision. More precisely, two-way ANOVA revealed a significant main effect for rotenone treatment (*F*(1, 23) = 42.231, *p* < 0.001) but not for currant supplementation (*F*(1, 23) = 1.891, *p* = 0.184). A significant interaction of rotenone treatment x Corinthian currant consumption has also been revealed (*F*(1, 23) = 10.059, *p* = 0.005). Further statistical analysis with Bonferroni’s post hoc comparison indicated a significant increase in both rotenone-treated groups (C-R: *p* < 0.001, CC-RC: *p* = 0.029). Additionally, a significant IL-1β immunodensity difference was demonstrated in the rotenone-treated group with currant diet in comparison with the corresponding rotenone-treated group on a conventional diet (R-RC: *p* = 0.004) (Figure 1(A_1_,A_2_)).

***SNpc*: **Two-way ANOVA indicated a statistically significant interaction between rotenone treatment and Corinthian currant consumption (*F*(1, 23) = 16.649, *p* < 0.001) regarding the immunoreactivity of IL-1β+ cells. Significant main effects for both rotenone treatment (*F*(1, 23) = 128.205, *p* < 0.001) and Corinthian currant consumption (*F*(1, 23) = 46.71, *p* < 0.001) were also observed (Figure 1(B_1_,B_2_)). Furthermore, a statistically significant increase in IL-1β+ cell density was demonstrated in both the rotenone-treated (*p* < 0.001) and rotenone-currant-treated (*p* < 0.001) groups compared to their respective controls. A slight but significant mitigation of IL-1β immunodensity was observed in the rotenone group supplemented with Corinthian currants compared to the rotenone cohort fed a standard diet (*p* < 0.001). In addition, we evaluated AChE immunoreactivity within the SNpc across all experimental groups (Supplementary Figure S2C). Two-way ANOVA revealed significant main effects for rotenone treatment (*F*(1, 23) = 519.47, *p* < 0.001) and Corinthian currant consumption (*F*(1, 23) = 9.685, *p* = 0.005), while no significant interaction (*p* = 0.186) between the two factors was detected (Figure 1(B_3_)). Specifically, a subsequent Bonferroni post hoc pairwise comparison showed that AChE+ cell density in the SNpc was significantly increased in both the Rotenone group (*p* < 0.001) and the Rotenone_currant group (*p* < 0.001), compared to their equivalent controls. No significant alterations were observed between the two control groups (*p* = 0.232). Conversely, a slight but significant attenuation of AChE+ cell density was noted in the rotenone-currant group compared to the rotenone-alone cohort (R-RC: *p* = 0.005; Figure 1(B_3_)). Next, we investigated whether the cytokine IL-1β is localized within AChE-expressing cells. Qualitative assessment revealed that a subpopulation of IL-1β+ nigral cells also expressed AChE (Figure 1C).

### 3.2. IL-1β Immunoreactivity Rotenone Treatment and Supplementary Diet with Corinthian Currants in Corticolimbic Brain Regions

**Figure 2:**
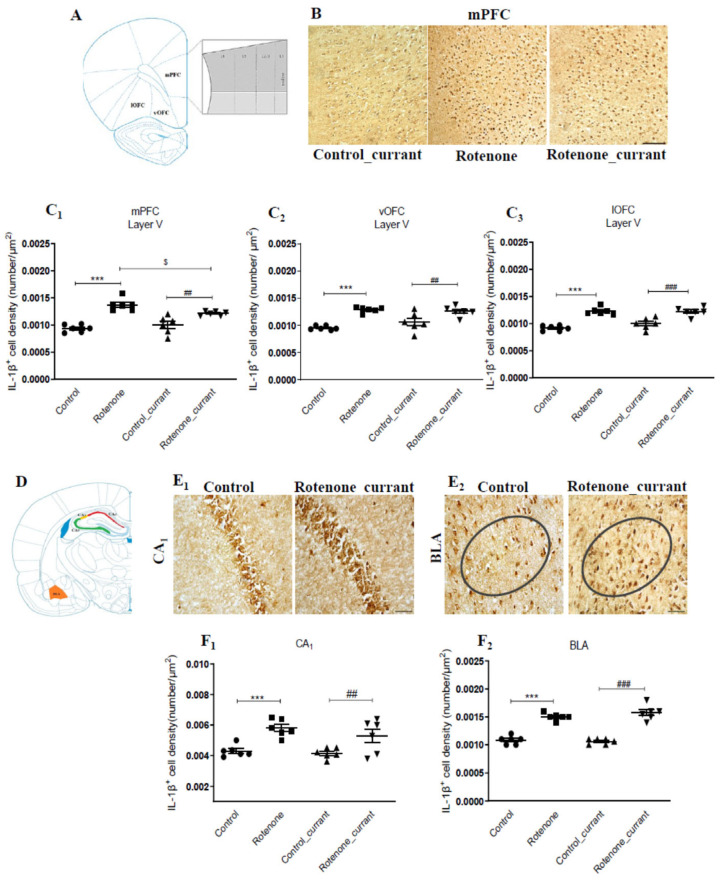
Effects of rotenone treatment and Corinthian currant consumption on IL-1β immunodensity in rat corticolimbic regions. (**A**) Schematic diagram shows the indicative coronal levels and layers of PFC. (**B**) Representative photomicrographs of control, rotenone, and rotenone_currant groups in mPFC. Scale bar = 0.05 mm. (**C_1–3_**) Scatterplots of IL-1β immunodensity in Layer V of the cortical subdivisions studied. (**D**) Schematic diagram showing the indicative coronal levels and subdivisions of the hippocampus (color coded) and basolateral amygdala, shown in orange (BLA). Representative photomicrographs of control and rotenone_currant groups in (**E_1_**) CA_1_ hippocampal region and (**E_2_**) BLA. (**F_1,2_**) Scatter dot plots of CA_1_ and BLA, respectively. Data are expressed as mean ± SEM, *n* = 6 per group. *** *p* ≤ 0.001 (Control-Rotenone), ^##^ *p* ≤ 0.01, ^###^ *p* ≤ 0.001 (Control_currant-Rotenone_currant), ^$^ *p* ≤ 0.05 (Rotenone- Rotenone_currant). The schematic diagrams are modified from [34].

***Prefrontal cortices*:** mPFC layers II/III IL-1β+ cell density showed a significant treatment (*F*(1, 23) = 46.725, *p* < 0.001) and interaction (*F*(1, 23) = 5.341, *p* = 0.030) effect. No significant currant effect (*F*(1, 23) = 1.477, *p* = 0.238) in mPFC layers II/III was determined. Specifically, an increase of IL-1β+ cells in mPFC layers II/III of the rotenone-treated groups with (*p* = 0.005) or without (*p* < 0.001) currant intake was observed compared to their equivalent controls (Figure 2A,B, Supplementary Figure S1). Similarly, in the output layer V of mPFC, two-way ANOVA revealed no significant currant (*F*(1, 23) = 1.105, *p* = 0.306), but significant treatment (*F*(1, 23) = 54.712, *p* < 0.001) and interaction (*F*(1, 23) = 6.079, *p* = 0.023) effects. Further analysis with Bonferroni’s post hoc comparisons revealed increased IL-1β+ cell densities in mPFC layer V of rotenone-treated rats with (*p* = 0.002) or without (*p* < 0.001) currant intake compared to their controls, respectively. Of note, a slight but significant decrease (*p* = 0.022) in the number of IL-1β+ cells was observed in rotenone-treated rats with a currant diet compared to the rotenone group consuming only the conventional food (Figure 2(C_1_)).

Subsequently, we examined IL-1β+ cell density in rat ventral and lateral orbitofrontal cortex (vOFC, lOFC). No significant interaction and currant effects in layers II/III of vOFC (interaction: *F*(1, 23) = 0.528, *p* = 0.476; currant: *F*(1, 23) = 0.097, *p* = 0.759) and lOFC (interaction: *F*(1, 23) = 1.960, *p* = 0.177; currant: *F*(1, 23) = 0.220, *p* = 0.644) were determined. However, two-way ANOVA revealed a significant simple treatment effect in layers II/III of vOFC (*F*(1, 23) = 26.958, *p* < 0.001) and lOFC (*F*(1, 23) = 16.017, *p* < 0.001). More specifically, a significant increase in IL-1β+ cells inof rotenone-treated rats without currant intake compared to their controls was determined in layers II/III of vOFC (*p* = 0.005) as well as lOFC (*p* < 0.001) (Supplementary Figure S1). In vOFC, IL-1β+ cells were increased (*p* < 0.001) in rotenone-treated rats following a currant diet as well. Similarly, no significant interaction and currant effects in layer V of vOFC (interaction: *F*(1, 23) = 2.675, *p* = 0.118; currant: *F*(1, 23) = 0.842, *p* = 0.370) and lOFC (interaction: *F*(1, 23) = 1.945, *p* = 0.178; currant: *F*(1, 23) = 1.443, *p* = 0.244) were determined. In vOFC and lOFC layer V, two-way ANOVA revealed that only the simple treatment (vOFC: *F*(1, 23) = 40.732, *p* < 0.001); lOFC: *F*(1, 23) = 72.636, *p* < 0.001) effect was significant, showing increased IL-1β+ cell densities in rotenone-treated rats with (vOFC: *p* = 0.003; lOFC: *p* < 0.001) or without (*p* < 0.001, for both cases) currant intake compared to their controls (Figure 2(C_2_,C_3_)).

***Hippocampus***: Two-way ANOVA indicated no significant interaction and currant effects in CA_1_ (interaction: *F*(1, 23) = 0.450, *p* = 0.510; currant effect: *F*(1, 23) = 1.488, *p* = 0.237, Figure 2(D,E_1_,F_1_)) and CA_2_ (interaction: *F*(1, 23) = 1.303, *p* = 0.267; currant effect: *F*(1, 23) = 0.883, *p* = 0.359) hippocampal regions. In contrast, significant treatment effects on IL-1β+ cell density of CA_1_ (*F*(1, 23) = 23.506, *p* < 0.001) and CA_2_ (*F*(1, 23) = 18.397, *p* < 0.001) were determined. Further analysis with post hoc comparisons in CA_1_ revealed increased IL-1β+ cell densities of rotenone-treated rats with (*p* = 0.008) or without (*p* < 0.001) currant intake compared to their controls (Figure 2(F_1_)). Of note, in CA_2_ a significant increase (*p* < 0.001) in IL-1β+ cells was observed in rotenone-treated rats without currant diet compared to their controls (Supplementary Figures S1D and S2B). Subsequently, we examined IL-1β+ cell density in the CA_3_ hippocampal region. Interestingly, IL-1β+ cell density in CA_3_ showed significant interaction (*F*(1, 23) = 6.633, *p* = 0.018), rotenone treatment (*F*(1, 23) = 30.402, *p* < 0.001) and currant (*F*(1, 23) = 12.474, *p* = 0.002) effects (Supplementary Figures S1E and S2B). Increased (*p* < 0.001) IL-1β+ cell densities were observed in the rotenone group compared with the control group. A significant decrease (*p* < 0.001) of IL-1β^+^ cells was observed in the CA_3_ hippocampal region of the rotenone-currant group compared with the rotenone group.

***Amygdaloid Complex***: In the basolateral amygdala (BLA) IL-1β+ cell density showed a significant rotenone treatment (*F*(1, 23) = 176.440, *p* < 0.001), but no significant currant consumption (*F*(1, 23) = 0.496, *p* = 0.489) nor interaction (*F*(1, 23) = 1.829, *p* = 0.191) effects. Bonferroni’s multiple comparisons showed increased IL-1β+ cell densities in the rotenone groups with (*p* = 0<001) or without (*p* < 0.001) currant intake compared to the equivalent control groups (Figure 2(E_2_,F_2_), Supplementary Figure S2B).

### 3.3. MAO-B Immunoreactivity Following Rotenone Treatment and Supplementary Diet with Corinthian Currants

**Figure 3:**
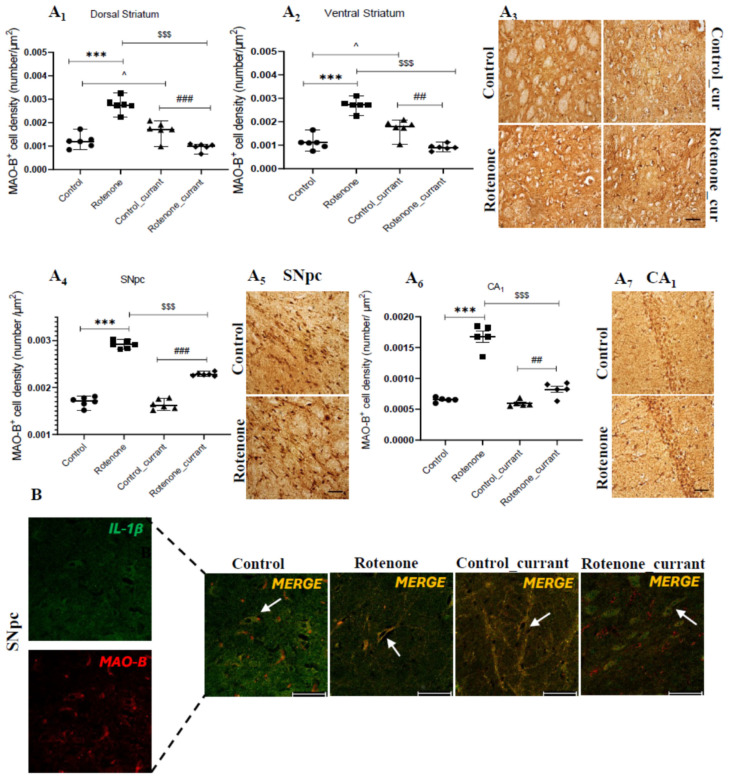
Effects of rotenone treatment and Corinthian currant consumption on MAO-B immunodensity. Scatter dot plots showing MAO-B+ cell density in rat (**A_1_**) Dorsal and (**A_2_**) Ventral Striatum, (**A_4_**) SNpc, and (**A_6_**) CA_1_ hippocampal subregion. (**A_3,5,7_**) Representative photomicrographs of MAO-B+ cells in the regions studied. Scale bar = 0.05 mm. Data are illustrated in scatter-dot plots and expressed as mean ± SEM, *n* = 5–6 per group. *** *p* ≤ 0.001 (Control-Rotenone), ^##^ *p* ≤ 0.01, ^###^ *p* ≤ 0.001 (Control_currant-Rotenone_currant), ^^^ *p* ≤ 0.05 (Control-Control_currant), ^$$$^ *p* ≤ 0.001 (Rotenone- Rotenone_currant (**B**) Representative confocal microphotographs showing the colocalization of IL1β+ (green) with MAO-B+ (red) cells in SNpc of control and rotenone-treated rats following conventional and supplementary Corinthian currant diet. White arrows show indicative double-labeled cells of IL-1β+ and MAO-B+. Scale bar = 0.05 mm.

***Striatum*:** In the dorsal striatum (Figure 3(A_1_,A_3_)), two-way ANOVA indicated simple main effects of treatment (*F*(1, 23) = 11.92, *p* = 0.002) and currant consumption (*F*(1, 23) = 30.302, *p* < 0.001) and an interaction between the factors (*F*(1, 23) = 87.692, *p* < 0.001). Subsequent analyses with Bonferroni’s post hoc test revealed a statistically significant increase in MAO-B+ cell density in rotenone-treated groups following conventional food intake (C-R, *p* < 0.001). Significant alterations were also observed following the comparison between the two rotenone-treated (R-RC: *p* < 0.001) groups. On the other hand, in the Ventral striatal subdivision (Figure 3(A_2_)), no simple effects were observed (treatment: *F*(1, 23) = 0.958, *p* = 0.339, currant: *F*(1, 23) = 3.865, *p* = 0.063), while an interaction between the two factors was evaluated (*F*(1, 23) = 25.809, *p* < 0.001). Similarly, in the ventral subdivision, increased MAO-B+ cell density was found in the rotenone-treated group consuming conventional food compared to controls (C-R: *p* < 0.001). Comparison between the two rotenone groups indicated a significant decrease in MAO-B+ cell density in the Rotenone_currant group (*p* < 0.001).

***SNpc*:** Two-way ANOVA revealed statistically significant main effects for rotenone treatment (*F*(1, 23) = 667.595, *p* < 0.001) and Corinthian currant consumption (*F*(1, 23) = 93.131, *p* < 0.001) as well as an interaction between the two factors (*F*(1, 23) = 63.765, *p* < 0.001). Subsequent post hoc analysis demonstrated a significant increase in MAO-B+ cell density in the rotenone-treated group (*p* < 0.001). Conversely, no significant differences were detected between the two control groups (C-CC: *p* = 0.253). Comparison between the two rotenone cohorts indicated a slight but statistically significant reduction in MAO-B+ cell density within the Rotenone_currant group (R-RC: *p* < 0.001, Figure 3(A_4_,A_5_)). Parallel to these quantitative changes, double immunofluorescence staining revealed that a subpopulation of IL-1β+ nigral cells was found to co-express MAO-B (Figure 3B).

***Hippocampus*:** As shown in Figure 3(A_6_,A_7_), two-way ANOVA revealed statistically significant main effects for both rotenone treatment (*F*(1, 19) = 136.969, *p* < 0.001) and Corinthian currant consumption (*F*(1, 19) = 73.615, *p* < 0.001), along with a significant interaction between the two factors (*F*(1, 19) = 55.428, *p* < 0.001). Subsequent statistical analysis with Bonferroni’s post hoc test demonstrated a significant increase in MAO-B+ cell density in the rotenone-treated group fed a standard diet compared to the control group (C-R: *p* < 0.001) and in the rotenone group supplemented with Corinthian currants compared with the control currant group (CC-RC: *p* = 0.008). Furthermore, a decrease in MAO-B+ cell density was observed in the rotenone-currant group compared with the rotenone-treated group (*p* < 0.001).

### 3.4. TNF-α Levels and Immunoreactivity Following Rotenone Treatment and Supplementary Diet with Corinthian Currants

**Figure 4:**
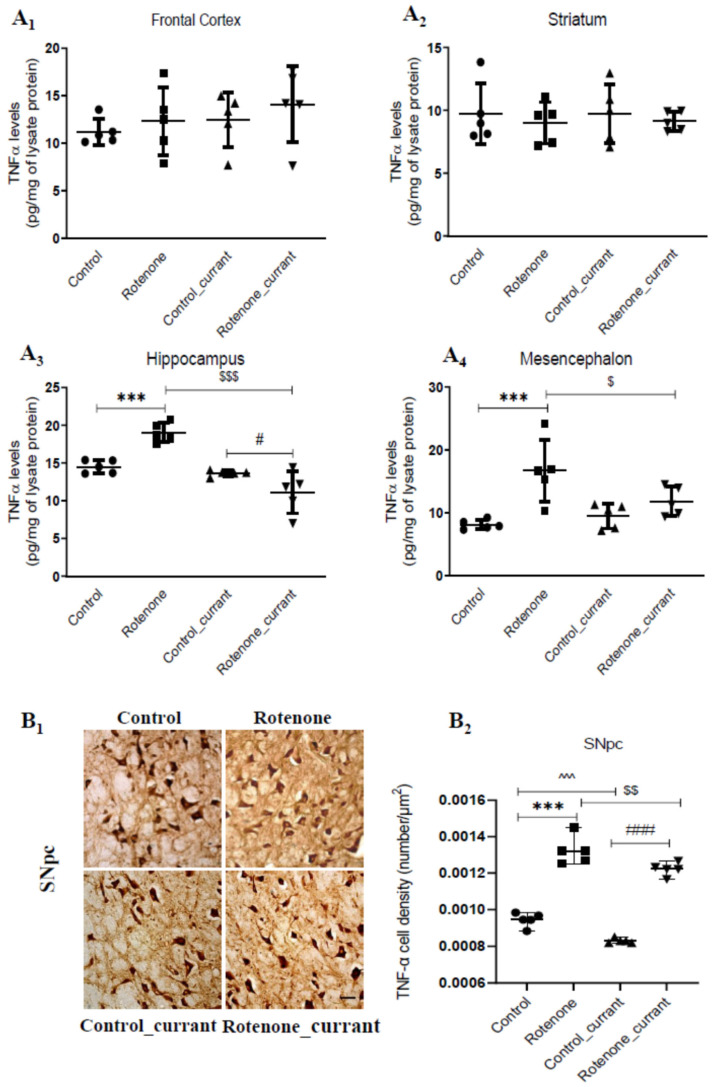
Levels of TNF-α in (**A_1_**) PFC, (**A_2_**) striatum, (**A_3_**) hippocampus, and (**A_4_**) mesencephalon. (**B_1_**) Representative photomicrographs of TNF-α^+^ cell density in the experimental groups in SNpc. Scale bar = 0.05 mm. (**B_2_**) Dot plot showing the effects of rotenone treatment and Corinthian currant consumption on TNF-α immunodensity in rat SNpc. Data are expressed as mean ± SEM, *n* = 5 per group. *** *p* ≤ 0.001 (Control-Rotenone), ^#^ *p* ≤ 0.05, ^###^ *p* ≤ 0.001 (Control_currant-Rotenone_currant), ^$^ *p* ≤ 0.05, ^$$^ *p* ≤ 0.01, ^$$$^ *p* ≤ 0.001 (Rotenone- Rotenone_currant) and ^^^ *p* ≤ 0.001 (Control-Control_currant).

***Modulation of TNF-α levels in Mesencephalon, Hippocampus, Striatum, and Frontal cortex*:** TNF-α levels in the aforementioned brain regions are shown in Figure 4. In the rat hippocampus (Figure 4(A_3_)), two-way Analysis of Variance indicated a significant main effect for Corinthian currant consumption (*F*(1, 19) = 38.260, *p* < 0.001) as well as a significant interaction between the two factors (*F*(1, 19) = 24.395, *p* < 0.001). Subsequent post hoc analysis using the Bonferroni test demonstrated a significant increase in TNF-α levels in the rotenone-treated group compared to its respective controls (C-R: *p* < 0.001). Furthermore, currant consumption induced a significant reduction in TNF-α levels within the rotenone- challenged cohort compared to the rotenone group fed a standard diet (R-RC: *p* < 0.001). Similarly, in Mesencephalon (Figure 4(A_4_)), Two-Way ANOVA revealed a significant main effect for rotenone treatment (*F*(1, 19) = 17.301, *p* < 0.001), and a significant interaction between rotenone treatment and Corinthian currant consumption (*F*(1, 19) = 5.543, *p* = 0.032). Post hoc analysis showed a significant increase in TNF-α levels in the rotenone group compared to the control (C-R: *p* < 0.001), whereas currant supplementation significantly attenuated TNF-α levels compared to the standard-diet rotenone group (R-RC: *p* = 0.019). In contrast, no significant effects for any of the factors studied were observed in the frontal cortex or the striatum (Figure 4(A_1_,A_2_)).

***Modulation of TNF-α immunoreactivity in SNpc*:** Following assessment of TNF-α levels by ELISA, we immunohistochemically evaluated its immunoreactivity in SNpc. Two-way ANOVA indicated significant main effects for both rotenone treatment (*F*(1, 19) = 329.164, *p* < 0.001) and currant consumption (*F*(1, 19) = 25.396, *p* < 0.001), without a significant interaction between the two factors (*F*(1, 19) = 0.187, *p* = 0.671). Specifically, TNF-α immunoreactivity was significantly elevated in both rotenone-treated groups, regardless of Corinthian currant supplementation (*p* < 0.001), compared with their respective controls. However, TNF-α immunoreactivity was significantly decreased in the currant-supplemented groups compared to their standard-diet counterparts (Control-Control_currant: *p* < 0.001, Rotenone- Rotenone_currant: *p* = 0.005), (Figure 4(B_1_,B_2_)). Furthermore, TNF-α expression in the rat hippocampus was evaluated via immunofluorescence labeling, as presented in Supplementary Figure S2A.

### 3.5. Co-Expression of Inflammatory Markers in Microglial Cells

**Figure 5:**
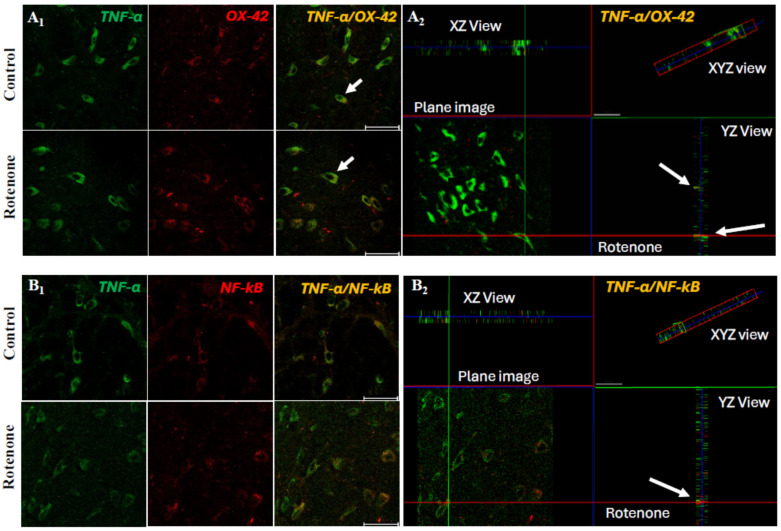
Double immunofluorescence (magnification ×63) showing the colocalization of TNF-α+ and (**A_1_**) OX-42+ or (**B_1_**) NF-κB+ cells in the SNpc of control and rotenone-treated rats. In the rotenone group, the vast majority of TNF-α+ (green) cells were also found to be OX-42+ (red) or NF-κB+ cells (red) in SNpc. The arrows show indicative double-labeled cells of TNF-α+ and OX-42+ or NF-κB+. Scale bar = 0.05 mm. Confocal 3D-orthogonal views from SNpc of the rotenone group showing the colocalization of TNF-α+ with (**A_2_**) OX-42+ or (**B_2_**) NF-κB+ cells. The green line indicates the X axis, the red line the Y axis, and the blue one the Z axis. Scale bar = 50 μm.

The co-localization of the pro-inflammatory cytokine, TNF-α, with the microglial marker CD11b (clone OX-42) and NF-κB within the SNpc (Figure 5) was evaluated to provide a qualitative and representative spatial assessment of these double-labeled subpopulations. Confocal 3D-orthogonal views verified a clear spatial overlap of the cytokine with the NF-κB signaling as well as with the OX-42, demonstrating its microglial localization. Although the quantification of the TNF-α-expressing cells is beyond the scope of the study, we estimated that the vast majority of TNF-a+ cell population was co-localized with the microglial marker within SNpc in both control groups (ranging 85–88%), while this percentage was found to be increased in the rotenone groups (89–91%).

**Figure 6:**
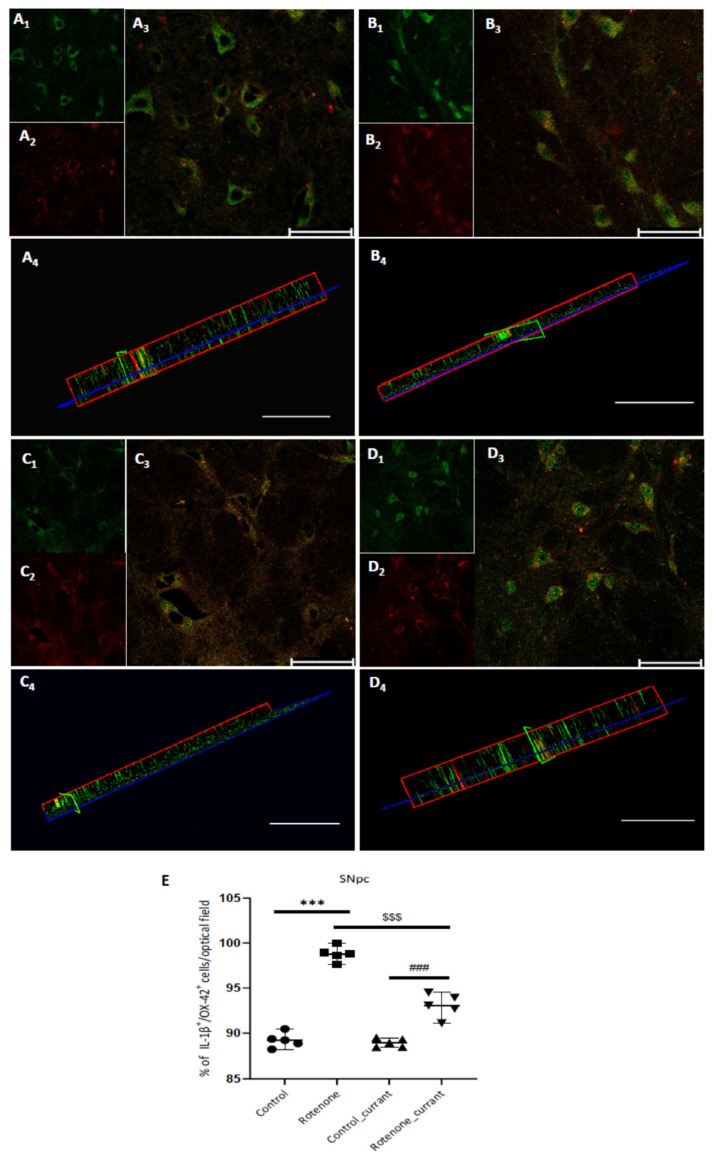
Double immunofluorescent labeling of IL-1β+ (green) with OX-42+ (red) cells in the SNpc of the (**A_1–3_**) Control, (**B_1–3_**) Rotenone, (**C_1–3_**) Control_currant, and (**D_1–3_**) Rotenone_currant groups. Scale bar = 0.05 mm. (**A_4_**,**B_4_**,**C_4_**,**D_4_**) Confocal 3D-orthogonal views from SNpc of control and rotenone groups showing the colocalization of TNF-α+ (green) with OX-42+ cells (red). The green line indicates the X axis, the red line the Y axis, and the blue one the Z axis. Scale bar = 50 μm. (**E**) Scatter- dot plot represents the percentage of IL-1β+/OΧ-42+. Data are expressed as mean ± SEM, *n* = 5 per group. *** *p* ≤ 0.001 (Control-Rotenone), ^###^ *p* ≤ 0.001 (Control_currant-Rotenone_currant), ^$$$^ *p* ≤ 0.001 (Rotenone- Rotenone_currant).

Subsequently, double-immunofluorescence staining was performed to evaluate the colocalization of the inflammatory cytokine IL-1β with the activated microglia marker OX-42 in SNpc (Figure 6). In the control group, 89.22% of IL-1β+ cells co-expressed OX-42. Following rotenone treatment, this percentage was significantly increased, reaching 98.79% (*p* < 0.001). Conversely, the percentage of IL-1β+/OΧ-42+ cells was significantly attenuated in the rotenone group with supplementary Corinthian currant diet compared to the rotenone-alone cohort (R-RC: 93.06%, *p* < 0.001), as graphically illustrated in Figure 6E.

## 4. Discussion

We have previously shown that thirty-eight days of dietary intervention with Corinthian currant, a fruit abundant in polar phenolic compounds [35], mitigated rotenone-induced dopaminergic cell loss and attenuated behavioral motor and anxiety deficits. Specifically, currant consumption partially rescued the TH and DAT expression, increasing the BDNF+ cell densities in the nigrostriatal pathway [25] as well as the accompanying serotoninergic (SERT, 5-HT) and noradrenergic (β_2_-ARs) dysfunctions in corticolimbic brain areas [32]. The present study continues our previous studies, focusing on the potential anti-inflammatory properties of dietary Black Corinthian currant in motor and non-motor brain areas, by evaluating the expression of pro-inflammatory cytokines IL-1β and TNF-α, utilizing the same rotenone rat model. The rationale of the present study is based on evidence suggesting that rotenone-induced oxidative stress activates microglia and astrocytes, increasing TNF-α, IL-1β, IL-6, iNOS, and COX-2, and driving dopaminergic neuron loss [31]. Importantly, our previous evidence on rotenone-induced toxicity and the dopaminergic protection of dietary Corinthian currants in the SNpc was replicated in the current study.

Recent studies suggest the role of neuroinflammation, a process involving the activation of glial cells and the increase of pro-inflammatory cytokines, in PD pathogenesis [7,36,37]. Our current findings demonstrate a significant increase in the pro-inflammatory cytokines, IL-1β and TNF-α, in motor (SNpc, striatum) as well as in limbic cognitive/emotional brain areas (frontal cortex, hippocampus, basolateral amygdala) in response to rotenone-induced toxicity. In line with our results, studies in PD patients and animal models revealed increased levels of IL-1β in serum, cerebrospinal fluid, striatum and midbrain [10,15,38,39,40]. Likewise, elevated protein levels of IL-1β and microglia activation have been observed in the ventral mesencephalon and SNpc of rodent models of PD [41,42].

Increasing evidence supports that diet and dietary components might significantly reduce the risk or alleviate the severity of PD-associated phenotype [43,44]. In vitro, cell-based, and animal studies have investigated the mechanisms of action of dietary polyphenols in promoting overall brain health [45]. Recent preclinical and clinical data propose their potential in reducing the advancement of neurological diseases and boosting cognitive abilities [46]. Specifically, PD inflammatory signaling pathways and oxidative stress have been suggested to be targeted by polyphenolic compounds [47,48,49] that act as potent antioxidants by scavenging ROS and increasing intracellular antioxidant enzymes such as superoxide dismutase and glutathione peroxidase [50,51]. Moreover, polyphenols potentially provide protection against neuroinflammation by the modulation of microglia and astrocytes [52]. Several polyphenolic compounds, including resveratrol, curcumin, and epigallocatechin gallate, have been shown to have inhibitory effects on microglial activation and the production of pro-inflammatory cytokines [53]. In agreement, the present study demonstrated that Corinthian currant dietary intervention attenuated the rotenone-induced overexpression of Il-1β and TNF-α in the nigrostriatal pathway, prefrontal cortex, and hippocampus. While the regional specificity of this anti-inflammatory protection cannot be definitively characterized, it may be linked to the differential neuroprotective efficacy of a polyphenolic diet across distinct brain areas. This variation could be attributed to differences in blood-brain barrier permeability, localized metabolite accumulation, regional antioxidant capacity, and regional density and phenotypic heterogeneity of microglia [54]. Particularly, the high density of microglial cells in the substantia nigra renders this region uniquely sensitive to the anti-inflammatory properties of polyphenols. Nonetheless, a limitation of the present study is the lack of evaluation of oxidative stress-related markers in the examined brain areas, given that oxidative stress is a well-known trigger for inflammatory pathways.

Polyphenols, present in plant-derived foods, including anthocyanins, flavonoids such as phenolic acids, quercetin, isoquercetin, resveratrol, and rutin, are widely distributed in fruits such as grapes and black dried currants [25,35,54]. Specifically, the dietary black Corinthian currant phenolic compounds, quercetin, isoquercetin, rutin, and isorhamnetin have been shown to cross the BBB, accumulating in the striatum, the mesencephalon, the hippocampus, the frontal cortex, and the cerebellum [25,26]. Interestingly, the rotenone-induced overexpression of IL-1β+ cell density was mitigated following thirty-eight days of Corinthian currant consumption in the SNpc, the striatum, the medial prefrontal cortex, and the hippocampus. In agreement, anti-inflammatory responses have been shown for flavonoids, a large group of polyphenolic compounds found in several fruits and vegetables [55], and for epigallocatechin, which increased cell viability, decreased apoptosis, and decreased the pro-inflammatory cytokines IL-1β and TNF-α [56,57,58].

In addition, the present study provided evidence on microglial localization (OΧ-42^+^) of IL-1β, TNF-α, and NF-κB expression in SNpc, supporting the idea that microglia-mediated neuroinflammation represents a target of currant polyphenolic anti-inflammatory action. Indeed, several lines of evidence suggest that inflammatory mediators such as TNF-α and IL-1β derived from microglia modulate the progression of neuronal cell death in PD [59]. Moreover, the inhibition of the nuclear transcription factor NF-κB is considered crucial for the regulation of inflammation [60], mediating the production of the effector cytokines TNF-α and IL-1β [61]. In fact, SNpc dopaminergic neurons are vulnerable to oxidative stress due to their high rates of catecholamine metabolism that drive the production of neuromelanin [62], which was shown to influence microglia activation through NF-κB in rodent microglial cell cultures [63]. In addition, ELISA experiments provided quantitative evidence of the modulation of TNF-α protein levels by rotenone treatment and Corinthian currant diet supplementation in the mesencephalon and hippocampus. However, further studies are needed to determine NF-kB signaling protein expression levels to reveal the exact mechanisms of the anti-inflammatory actions of currant diet supplementation. Taken together, our data support the idea that microglia activation induced by rotenone treatment results in the production of the pro-inflammatory cytokines IL-1β and TNF-α, which may lead to dopaminergic cell loss in the SNpc and the dopaminergic deficit in the striatum [25]. While microglia display several phenotypic profiles that may be affected by the local microenvironment and other factors [64], in PD, is known to exist in two phenotypic profiles: the neurotoxic, which is induced to release pro-inflammatory cytokines and chemokines, resulting in neurotoxicity, and the neuroprotective microglia, which regulate the neurotoxic function by releasing anti-inflammatory cytokines and chemokines, in order to restore brain homeostasis [65,66]. In the present study, specifically activated microglia, as evidenced by OX-42+ cells which co-express IL-1β, were upregulated by rotenone treatment. Moreover, the percentage of OX-42+/IL-1β+ profiles was significantly decreased by currant supplementation. Additional double-labeling studies would precisely identify the microglial population expressing TNF-α and the NF-κB transcription factor.

Similarly, dietary supplementation with currant partly reversed the increases in AChE+ and MAO-B+ cell densities observed in the rotenone-treated groups. Increased MAO activity is directly connected to neurological disorders such as depression and anxiety, as well as PD [67]. Moreover, MAO-B has been implicated in neuronal death in neurodegenerative diseases via reactive astrogliosis [68,69], while inhibitors of MAO-B restore dopamine levels and are used to treat PD [70]. In the present study, rotenone treatment significantly increased MAO-B+ cell densities in SNpc, striatum, and hippocampus, but a complementary diet with black currant reversed these increases. In support, a previous study showed that supplementation with blackcurrant extract almost completely inhibited blood platelet MAO-B activity [71]. Similarly, in rat striatum, tocopherol attenuated rotenone-induced elevated MAO-B activity [72]. Natural products consisting of selective and reversible inhibitors of MAO-B, such as phenolic compounds, may prove to be important therapeutic approaches for treating pathophysiological consequences of monoamines’ metabolism [73]. Furthermore, our study provided evidence on a modulatory role of currant dietary supplementation in nigral AChE expression. Specifically, in parallel to the upregulation of pro-inflammatory cytokines and MAO-B expression, rotenone treatment increased AChE+ cell density in SNpc, while currant supplementation mitigated this increase. Interestingly, AChE has been proposed to have a non-classical function in immune cells. Beyond its role in degrading acetylcholine—a neurotransmitter critical for memory—elevated AChE levels are also implicated in promoting neuroinflammation. Interestingly, AChE inhibitors, which are commonly used to treat cognitive symptoms in Alzheimer’s disease, have beneficial effects in Parkinson’s disease by reducing neuroinflammation and improving motor function [74]. In fact, pro-inflammatory responses may be modulated by acetylcholine activation of α7 nicotinic receptors (α7nAChR) signaling in macrophages [75] Moreover, acetylcholine regulates microglial activation, inhibiting TNF-α release in murine-derived microglial cells expressing α7nAChR [76]. In support, AChE inhibitors down-regulate the expression of pro-inflammatory cytokines such as IL-1β and TNF-α in Alzheimer’s disease patients [77]. Similarly, LPS-induced neuroinflammation upregulated AChE enzymatic activity via the NF-κB pathway in a cultured microglial cell line [78]. Accordingly, rotenone-induced upregulation of Il-1β expression coincides with AChE increases in SNpc, in agreement with their observed colocalization. While currant dietary supplementation attenuated AChE expression, the precise mechanisms of neuroprotection and the cellular origin of AChE remain to be fully elucidated, as both astrocytes and neurons may be the source of acetylcholine controlling microglial inflammation. Taken together, our results suggest that a complementary diet with black Corinthian currant has significant anti-inflammatory and neuroprotective effects in the brain areas of a PD rat model, primarily by downregulating proinflammatory cytokines, MAO-B, and AChE expression.

## 5. Conclusions

Our findings indicate that dietary supplementation with polyphenol-rich black Corinthian Currant has the potential to act as an anti-inflammatory agent in a rat rotenone PD model. Specifically, the currant-enriched diet attenuated the rotenone-induced upregulation of glial pro-inflammatory cytokines IL-1β and TNFα within motor and corticolimbic brain regions. In addition, currant consumption mitigated nigral degeneration, evidenced by the reduction of MAO-B and AChE expression. Collectively, our data support the neuroprotective properties of black Corinthian currant on brain inflammatory processes, highlighting its potential to ameliorate PD degeneration.

## Data Availability

The original contributions presented in this study are included in the article/Supplementary Materials. Further inquiries can be directed to the corresponding author.

## Supplementary Materials

The following supporting information can be downloaded at: https://www.mdpi.com/article/10.3390/antiox15070906/s1, Figure S1: Effects of rotenone treatment and Corinthian currant consumption on IL-1β+ cells in Layers II-III of Frontal cortices, CA2 and CA3 hippocampal sub-regions; Data are expressed as mean ± SEM, n = 6 per group. ** *p* ≤ 0.01 *** *p* ≤ 0.001 (Control-Rotenone), ^#^ *p* ≤ 0.05, ^##^ *p* ≤ 0.01, ^###^ *p* ≤ 0.001 (Control_currant-Rotenone_currant), ^$^ *p* ≤ 0.05, ^$$$^ *p* ≤ 0.001 (Rote-none- Rotenone_currant). Figure S2: Fluorescent immunolabelling of TNF-α^+^ and IL-1β^+^ cells in hippocampal sub-regions and AChE immunolabelling in SNpc.

## Abbreviations

The following abbreviations are used in this manuscript:

AChE	Acetyl-cholinesterase
BBB	Blood–Brain Barrier
BLA	Basolateral Amygdala
MAO-B	Monoamine oxidase-B
NF-kB	Nuclear Factor kappa-light-chain-enhancer of activated B cells
PD	Parkinson’s Disease
PFC	Prefrontal cortex
SNpc	Substantia Nigra pars compacta
TNF-α	Tumor necrosis Factor alpha
