# Supplementary figures and images for: Neuroprotective Effects of Polyphenol-Rich Corinthian Currant Against a Rotenone Parkinson’s Disease Model: Mitigation of MAO-B and Pro-Inflammatory Cytokines Upregulation in Motor and Limbic Brain Regions
Source: Antioxidants (Basel). 2026 Jul 22;15(7):906. doi: 10.3390/antiox15070906 (PMC13404455; doi:10.3390/antiox15070906)

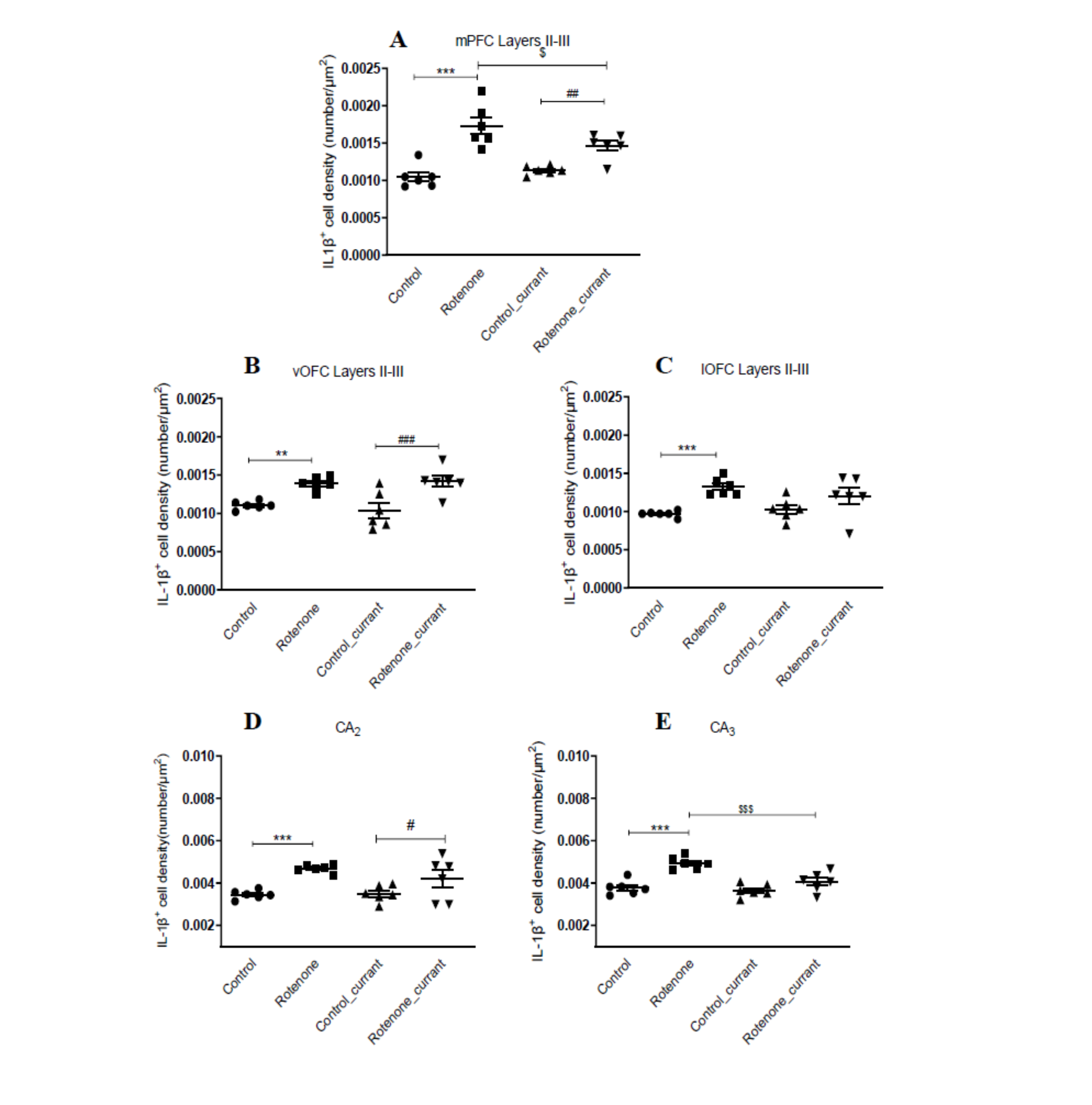

Supplement: Supplementary file 1 [file antioxidants-15-00906-s001.zip › SUPPLEMENTARY FIGURES1.tif]

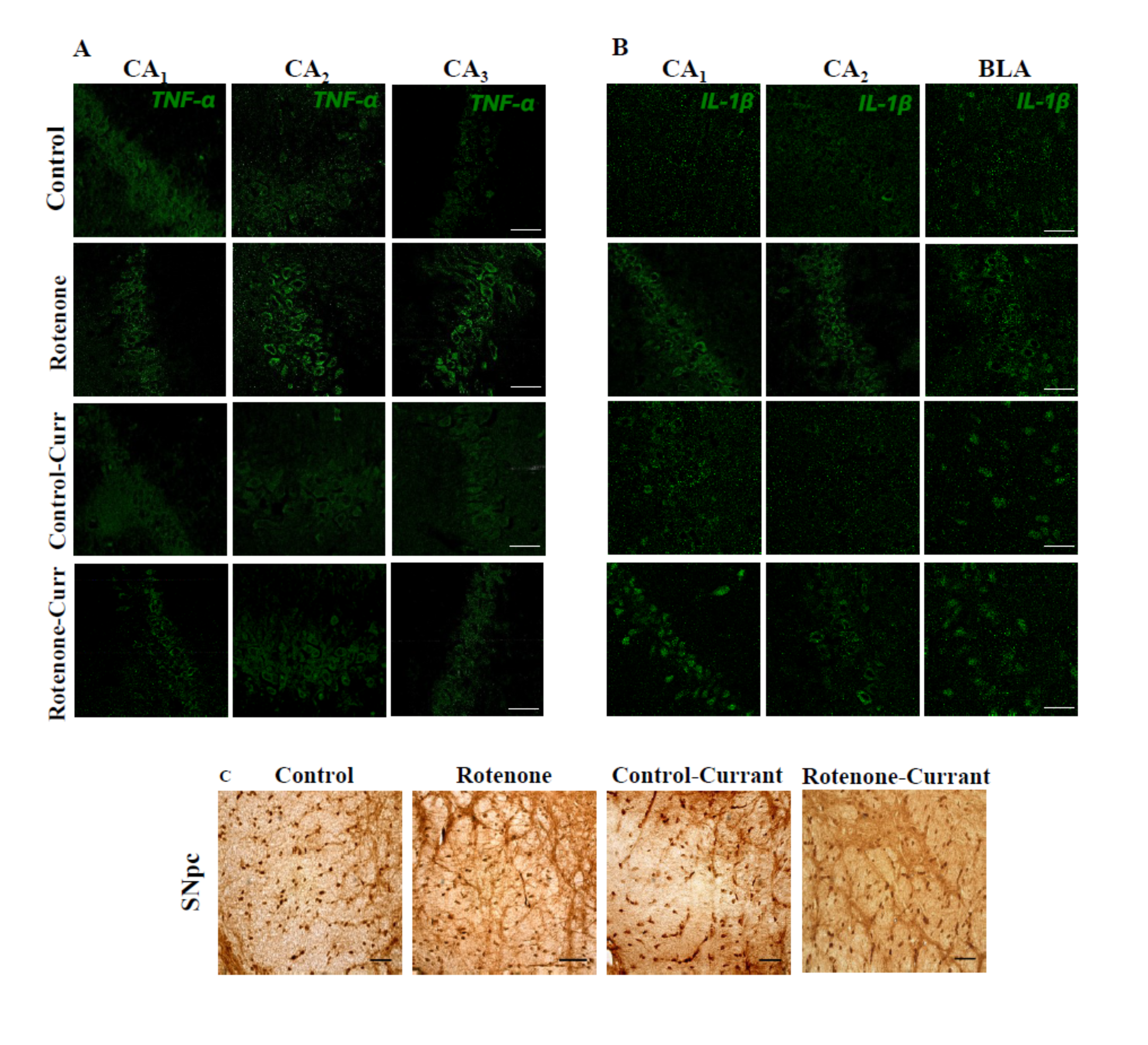

Supplement: Supplementary file 1 [file antioxidants-15-00906-s001.zip › SUPPLEMENTARY FIGURES2.tif]
